# A Novel Integration of IF-DEMATEL and TOPSIS for the Classifier Selection Problem in Assistive Technology Adoption for People with Dementia

**DOI:** 10.3390/ijerph19031133

**Published:** 2022-01-20

**Authors:** Miguel Angel Ortíz-Barrios, Matias Garcia-Constantino, Chris Nugent, Isaac Alfaro-Sarmiento

**Affiliations:** 1 Department of Productivity and Innovation, Universidad de la Costa CUC, Barranquilla 081001, Colombia; ialfaro1@cuc.edu.co; 2 School of Computing and Mathematics, Ulster University, Jordanstown BT37 0QB, UK; m.garcia-constantino@ulster.ac.uk (M.G.-C.); cd.nugent@ulster.ac.uk (C.N.)

**Keywords:** technology adoption, classifier, Intuitionistic Fuzzy Sets (IFS), Decision Making Trial and Evaluation Laboratory (DEMATEL), Technique for Order of Preference by Similarity to Ideal Solution (TOPSIS), multi-criteria decision making (MCDM), People with Dementia (PwD), public health

## Abstract

The classifier selection problem in Assistive Technology Adoption refers to selecting the classification algorithms that have the best performance in predicting the adoption of technology, and is often addressed through measuring different single performance indicators. Satisfactory classifier selection can help in reducing time and costs involved in the technology adoption process. As there are multiple criteria from different domains and several candidate classification algorithms, the classifier selection process is now a problem that can be addressed using Multiple-Criteria Decision-Making (MCDM) methods. This paper proposes a novel approach to address the classifier selection problem by integrating Intuitionistic Fuzzy Sets (IFS), Decision Making Trial and Evaluation Laboratory (DEMATEL), and the Technique for Order of Preference by Similarity to Ideal Solution (TOPSIS). The step-by-step procedure behind this application is as follows. First, IF-DEMATEL was used for estimating the criteria and sub-criteria weights considering uncertainty. This method was also employed to evaluate the interrelations among classifier selection criteria. Finally, a modified TOPSIS was applied to generate an overall suitability index per classifier so that the most effective ones can be selected. The proposed approach was validated using a real-world case study concerning the adoption of a mobile-based reminding solution by People with Dementia (PwD). The outputs allow public health managers to accurately identify whether PwD can adopt an assistive technology which results in (i) reduced cost overruns due to wrong classification, (ii) improved quality of life of adopters, and (iii) rapid deployment of intervention alternatives for non-adopters.

## 1. Introduction

As life expectancy has been increasing globally due to medical advances and improvements in the quality of life of people in later stages of life, age-related diseases such as dementia have also been increasing. The growing number of People with Dementia (PwD) [1] and the insufficient number of formal and informal carers [2] for them have resulted in (i) inadequate care and lower quality of life for PwD, and (ii) increased burden and stress for carers. Nonetheless, technology solutions have been used to improve the quality of life of PwD, to support the work of carers, and to provide more personalized types of treatment. Assistive Technology-based Solutions (ATS) [3] have been commonly prescribed as a form of non-pharmacological treatment to PwD to help them complete everyday activities whilst maintaining a level of independence, yielding health and social benefits for them. Some of the areas in which ATS provide support include memory, mobility, indoor and outdoor safety, independence, and socializing. Technological advances in terms of devices that can be useful for supporting PwD and connectivity in the context of Internet of Things (IoT) are constantly evolving and represent a useful technological implementation to support the work of carers to improve the quality of life of PwD. In both cases of device and connectivity advances, capabilities are increasing and costs are decreasing.

Although the benefits of using ATS to improve the quality of life of PwD have been demonstrated, low adoption of ATS has represented an obstacle in their more widespread use and in obtaining their potential benefits over time. The main causes for low adoption of technology-based solutions to assist PwD may be that they are not the most adequate for particular individuals. In many instances, the implementation of certain ATS is not user-friendly enough and/or disliked by the PwD, which results in the early use of the technology to gradually decrease until it is discarded. In addition to not providing benefits to the quality of life of PwD, the rejection of ATS also impacts health services and relatives of PwD in terms of time and financial resources spent in the unsuccessful attempts of adopting the technology. The identification of needs and preferences of PwD for a personalized assessment in the use of ATS is essential for their adoption and use, which can result in a successful treatment. It is desired that ATS maximize the benefits for the quality of life of PwD while also being cost-effective. In the context of personalized health interventions, it is necessary to identify the most significant features of potential users in the form of personal profiles and individual preferences to obtain the most benefits of ATS interventions. These significant features are then used to identify potential adopters and non-adopters of ATS amongst prospective users for (i) avoiding cost implications, (ii) improving the quality of life of adopters, and (iii) finding adequate intervention alternatives for non-adopters. It is expected that over time, the use of these significant features will allow the generation of user profiles of potential adopters and non-adopters in a more rapid and accurate manner. In most health organizations around the world, the most ethical and humane approach followed is to offer potential treatments to all the sufferers of a condition; in our case, providing assistive technology to PwD. It is acknowledged that not all PwD will adopt the assistive technology, so having a better way to identify which ones are more likely to adopt it will reduce costs (staff, equipment, drugs, and training) and will provide benefits to the PwD willing to adopt the technology in support of, or instead of, using pharmacological treatments.

Adoption model studies, also known as classifiers, are typically used to support the process of identifying adopters and non-adopters of ATS by providing insights on how successfully a PwD will adopt a certain ATS based on their input criteria from different domains. The classifier selection problem in Assistive Technology Adoption refers to selecting the classification algorithms that have the best performance in predicting the adoption of technology, and is often addressed through measuring different single performance indicators. Satisfactory classifier selection can help in reducing time and costs involved in the technology adoption process. The adequate selection of significant features greatly affects the performance of the classifiers used for adoption model studies. Therefore, this paper aims to select the most appropriate classification algorithms to support the technology adoption task based on the different criteria available. Multiple-Criteria Decision-Making (MCDM) methods [4] are used to assign weights to the criteria and to rank the classifiers considered. These methods are appropriate tools in supporting developers, healthcare professionals, and practitioners in the selection of the most suitable classifiers for ATS in PwD. This paper presents a novel approach that integrates Intuitionistic Fuzzy Sets (IFS) [5], Decision Making Trial and Evaluation Laboratory (DEMATEL) [6], and the Technique for Order of Preference by Similarity to Ideal Solution (TOPSIS) [7] for the classifier selection problem. IF-DEMATEL is first used to estimate the criteria and sub-criteria weights considering uncertainty, and then to help to evaluate the interrelation among classifier selection criteria. TOPSIS is applied to generate an overall suitability index per classifier so that the most effective ones are selected. The integration of these methods is novel in selecting classifiers for supporting technology adoption in PwD. In fact, we aim to network with people from public health organizations to obtain their judgments regarding the importance of criteria influencing this decision. Note that the merger of these two methods has already been successfully adopted in other fields [8,9].

The combination of these single methods in this application targets the following objectives:

▪ The IF-DEMATEL technique allows for detecting and measuring potential cause-effect interrelations among decision criteria involved in technology adoption. Its inclusion lays the groundwork for the identification of the main drivers underpinning the effective design, development, and implementation of classification algorithms in the real world. In addition, it considers the vagueness and uncertainty inherent in human judgments, an aspect of paramount importance to take into account from the participation of different stakeholders whose expectations are desired to be fully incorporated into the classifier selection model [10,11]. Another contribution from this method is the possibility to estimate the importance of conflicting criteria and sub-criteria with respect to the goal; in this case, the identification of the most suitable classifier supporting technology adoption in PwD.
▪ It is straightforward to apply the TOPSIS method in the wild (physical, environmental, and organisational elements) and it is employed for ranking classifier alternatives based on a data-driven approach generating a closeness coefficient (in this case, the suitability index). One of the main weaknesses to be overcome in this technique is the allocation of weights that are originally assigned randomly [7,12]. The inclusion of IF-DEMATEL tackles this disadvantage by providing a solid mathematical foundation considering interdependences and feedback among the decision elements.

The proposed approach is validated using a real-world study (five criteria, 16 sub-criteria, and five alternative classifiers) concerning the adoption of a mobile-based reminding solution by PwD. The criteria are decision elements directly influencing the classifier selection problem in Assistive Technology Adoption (e.g., performance, applicability, and replicability). These elements are subdivided into more specific categories called “sub-criteria”; for instance, the criterion “applicability” has been partitioned into two sub-criteria: “Interpretability” and “Ease of comprehension by non-experts”. Thereby, a complex problem is addressed through a hierarchy comprising of a set of interconnected sub-problems.

The main motivations for the case study presented are in terms of cost–benefit: (i) to use public health resources in the most effective way, and (ii) to provide the most health benefits to PwD that are willing to adopt a non-pharmacological treatment. In addition to supporting the investigation of ATS adoption by PwD, this approach also investigates issues found in the implementation of technologies in the wild. This work evidences the factors that need to be primarily addressed when developing the classifiers and provides further recommendations to improve their suitability upon scaling up. Our classifier selection approach can be applied to other areas, such as stock index price movement prediction, intruder detection systems, bankruptcy prediction, and chemical fertilizer selection.

The remainder of the paper is organized as follows: Section 2 presents the related work in ATS for PwD focusing on the IF, DEMATEL, and TOPSIS methods. Section 3 outlines the proposed methodology. Section 4 presents a case study that considers the adoption of a mobile-based reminding solution by PwD. Conclusions and future work are presented in Section 5.

## 2. Related Work

### 2.1. A Background on Assistive Technology Solutions (ATS) for PwD

The use of ATS in health has been covered extensively with general approaches [13,14,15] and with approaches focusing on specific health problems such as visual impairment [16], disabilities [17,18], and dementia [3,19,20,21,22,23]. This section provides an insight into recent relevant work in ATS for PwD, which has helped to identify similar approaches to the one presented in this paper, to which to compare to and learn lessons from. This section also presents how our approach is positioned within the literature and how it contributes in the context of ATS in health.

As the prevalence of dementia is expected to increase in the near future, it is important to identify the most adequate ATS to alleviate the burden of patients and caregivers while taking into account the range of cognitive abilities that impair the adaptation and use from PwD. It is therefore of paramount importance to select classifiers or predictive models that can discriminate between adopters and non-adopters of these technologies. In this regard, Zhang et al. [24] investigated the use of a predictive adoption model for a mobile phone-based video streaming system for PwD, which considered features such as users’ ability, preferences, and living arrangements. The predictive models developed were based on seven classification algorithms and were evaluated using multiple criteria of model predictive performance, robustness, bias towards two types of errors, and usability. In the results reported by Zhang et al. [24], the predictive model trained using the kNN (k-Nearest Neighbor) classification algorithm was the best to support the adoption of assistive technology with a prediction accuracy of 0.84 ± 0.0242. The technology adoption problem was also addressed by Chaurasia et al. [20] who presented an ATS for PwD in the form of the Technology Adoption and Usage Tool (TAUT) reminder app to identify a subset of relevant features that could then be used to improve the accuracy of technology adoption prediction. The 31 features considered for the data analysis were from the Cache County Study on Memory and Aging (CCSMA) and included features such as range of age, gender, education level, and health condition. Chaurasia et al. [20] reported that although features related to the background of PwD (such as job or education level) can have an impact on the user’s technology adoption, other features related to the dementia condition (such as genetic markers and comorbidity) could decrease the adoption of assistive technology. The best prediction model was obtained using the kNN classification algorithm, with an average prediction accuracy of 92.48% when tested on 173 participants. The findings of Chaurasia et al. [20] are that relevant features can provide an insight into the adoption of ATS by PwD. In a continuation to their previous research, Chaurasia et al. [21] presented the analysis and fusion of two datasets in the context of modeling mobile-based technology by PwD. As in their previous work, Chaurasia et al. [21] used the TAUT reminder app and extracted more detailed information resulting from the fused datasets, which included the background, psychosocial, and medical history of the 173 participants involved. This set of features was used to develop a technology adoption model, which obtained the following best prediction accuracy results using classification algorithms: 99.41% with the kNN and 94.08% with Neural Networks. These classification results were an improvement from the best classification result presented in their previous work [20], which was 92.48% using the kNN classification algorithm. Chaurasia et al. [21] reported that better results were obtained using a combination of psychosocial and medical data from the participants, and noted that it is preferable that medical data are provided from reliable medical sources rather than self-reported by the participants. Another interesting approach considering a different ATS was presented by Cruz-Sandoval et al. [23], who investigated the adoption process of wearable fitness trackers by PwD in a Cognitive Simulation Therapy (CST) enabled by a conversational social robot. In particular, Cruz-Sandoval et al. [23] framed the issues regarding the wearable fitness trackers adoption using an extension of the Unified Theory of Acceptance and Use of Technology (UTAUT) and based their recommendations on the Dementia Design Considerations for Smart Home Technologies targeting four domains (cognitive decline, physical decline, social, and development). Cruz-Sandoval et al. [23] reported that the results obtained provide evidence of the feasibility of the sustained use of activity trackers by PwD during prolonged periods. In light of the literature, it is evident that technology adoption in PwD depends on different features or predictors, and not only the simple preference or opinion of the primary carers.

It is also important to point out the key factors to take into consideration when deploying ATS, in addition to the barriers that need to be overcome for ensuring their effective adoption by PwD. These are valuable insights supporting the development of classifier selection models that guide practitioners to choose the best algorithm in a particular application context. In this respect, Øksnebjerg et al. [25] presented a review to investigate user involvement, dissemination, and adoption of assistive technology by PwD. The papers included in the review were eleven papers from eight studies. Øksnebjerg et al. [25] reported that none of the studies considered dissemination, user involvement varied from extensive to none, and methods for adoption also varied and only targeted prototype testing. Some of the adoption methods reported from the reviewed papers were individual introduction, individualized program, audio-based guidance included in an app, a training session and manual for caregivers, and an individualized training session for both participant and caregiver. In a similar vein, Øksnebjerg et al. [26] merged various methods to promote the ability to cope with, and the adoption of, assistive technology for PwD. The pilot intervention was in the form of sessions (individual and group) for PwD, and used a tablet app called ReACT (Rehabilitation in Alzheimer’s disease using Cognitive support Technology). The ReACT app addressed individual goals and more general self-management approaches. The study, comprising 19 PwD, reported positive attitude from PwD, high attendance, and that the program used and their intervention can be an effective way to promote ATS adoption by PwD. Identifying the obstacles for ATS adoption in PwD is pivotal to upgrading the robustness of the classifier selection models. In this sense, Fotteler et al. [15] investigated the obstacles of using assistive technologies for healthy older adults. Although in this case the participants were not PwD, it is interesting how different topics can influence older adults’ adoption of assistive technologies. The results reported in this work indicate that the participants in the study emphasized the need for repeated training, a user-friendly and accessible design, and clarity on cost reimbursement or insurance coverage. From their results, the authors concluded that lack of knowledge regarding assistive technologies is a greater barrier in the acceptance by older people.

### 2.2. Technology Adoption in PwD from an MCDM Perspective

Despite the above-mentioned efforts, the use of classifiers for supporting technology adoption in PwD has been only skewed to the performance domain. Transferring ATS, however, requires consideration of several aspects from the public health sector, potentially hindering the correct allocation of these solutions to PwD while ensuring effective scale-up. The classification models are expected to be congruent with this multi-criteria healthcare context and should be therefore carefully selected prior to use in the practical scenario. As there are several conflicting criteria and sub-criteria to consider, in addition to diverse classifier alternatives, this becomes an MCDM problem. MCDM, as an operational research discipline, deals with very complex decision problems by establishing a structure of criteria, sub-criteria, alternatives, and decision makers’ preferences [27]. MCDM helps managers to select the best choice from a set of feasible options (in this case, classifiers) in the presence of various conflicting criteria. MCDM is classified into two major components [28]: Multi-attribute Decision Analysis (MADA) and Multi-objective Decision Analysis (MODA) methods. MADA, the methodological branch explored in this paper, copes with a limited set of alternatives as evidenced in our case study.

A related contribution in this direction can be found in [3], where a multi-criteria framework was proposed to select the most adequate classification algorithm in an ATS adoption process. This framework is based on the integration of a five-phase methodology based on the Fuzzy Analytic Hierarchy Process (FAHP) and the Technique for Order of Preference by Similarity to Ideal Solution (TOPSIS): FAHP-TOPSIS. The approach was validated using the case study of a mobile-based self-management and reminding solution for PwD. FAHP was used to determine the relative weights of criteria (performance, usability, scalability, flexibility, and design) and sub-criteria under uncertainty. TOPSIS was used to rank the seven classification algorithms considered. Ortiz-Barrios et al. [3] reported that the best results to support the adoption of assistive technology were obtained using the kNN classification algorithm, with a closeness coefficient of 0.804. In this case, the most important criterion for classification selection was scalability. Following this, the approach presented by Ortiz-Barrios et al. [29] extended our previous work [3] by considering the VIKOR method instead of TOPSIS to rank different classification algorithms. FAHP was used to calculate the criteria and sub-criteria weights under certainty and then VIKOR was implemented to rank the classifiers. The case study used to validate the approach was the mobile-based self-management and reminding solution considered in [29]. In terms of the ATS for PwD, results revealed that the most important criteria were “Easiness of interpretation” and “Handling of missing data”. The classification algorithms that obtained the best results to support ATS adoption in PwD were Support Vector Machines (SVMs) and AB.

A range of single MCDM methods, both single and hybrid, can be alternatively used for the classifier selection problem. In this case, however, a blend of two or more methods (hybrid) is preferred over single techniques considering the decision-making scenario evidenced in healthcare technology adoption: (i) assessment of criteria weights under uncertain information, (ii) appraisal of interrelations and feedback within the network, (iii) calculation of classifier suitability index, and (iv) delineation of improvements for the design/development process. Furthermore, hybridization is a current trend in the MCDM domain because [30,31]: (i) it tackles the limitations that single approaches hold in their original structure, and (ii) it allows for merging quantitative and qualitative data, which is advantageous considering the nature of the factors affecting technology adoption in PwD. This approach is strengthened when incorporating the Intuitionistic Fuzzy Sets (IFSs) due to their capability for representing the ambiguity and vagueness of decision makers when performing the judgments [32,33]. That is, this type of fuzzy set is utilized when the experts are unsure about their judgments [34] and is therefore useful to deal with complex real-life MCDM problems identified in technological applications such as the one presented in this paper. An example of this research direction is illustrated by [35], who implemented interval-valued IF-AHP to support a new digital service quality model in the Turkish aviation industry. Another interesting work was authored by [36] to identify the most influential dimensions and criteria for Smart City Development (SCD) in the Moroccan context. Specifically, IFSs were used to model the hesitation of decision makers when deciding on the importance of criteria and sub-criteria, an aspect commonly observed in practical cases. IFSs have been also widely applied in the healthcare context for underpinning various decision-making scenarios, modeling the response to the COVID-19 pandemic [37], hospital performance evaluation [38], hazardous waste management [39], and patient safety management [40]. Taking into account the above implications, it was decided to implement a hybrid fuzzy method in this work.

Considering the above, it is pivotal to identify the combination of MCDM techniques responding to the above-described decision-making scenario in a rapid and effective manner. IF-DEMATEL is proposed to deal with the calculation of criteria priorities [41] considering uncertainty, whereas the interdependence and feedback is assessed in the decision-making model. IF-DEMATEL can address these methodological requirements because: (i) it utilizes impact digraphs facilitating the identification of one- or bi-directional interrelations among decision criteria [42], (ii) it allows for pinpointing the dispatching and receiving factors that enable developers to craft targeted interventions, increasing the suitability of the algorithms in the clinical context, (iii) it incorporates the hesitancy function and the membership and non-membership degrees to represent the unfavorability, favorability, and the neutrality that decision-makers may experience when analyzing the influence of each factor and sub-factor, (iv) it promotes the participation of different stakeholders involved in the classifier design, development, and implementation phases via group decision-making, (v) it elicits the priorities of criteria and sub-criteria based on relation and prominence measures [6], and (vi) it is easy to implement in the healthcare sector if suitable friendly data-gathering instruments are adopted by the project leaders. The Analytic Network Process (ANP) or its intuitionistic fuzzy version (IF-ANP) can also evaluate network relationships among criteria; however, it has been found to be time-consuming in large-sized models due to the rigorous estimation of composite priorities. Furthermore, the premise of equal priorities for clusters when deriving the weighted supermatrix is not valid in the practical scenario where these scores may vary [43,44]. By comparison, TOPSIS is suggested to cope with the ranking of classifier alternatives [45] and the definition of improvement strategies in view of: (i) the resulting closeness coefficient that can be assumed as an index denoting the appropriateness of each classifier, (ii) the easy identification of suitable algorithms based on the index proposed in the previous point, and (iii) the calculation of Euclidean distances to both ideal and anti-ideal solutions [31,46,47], which permits the identification of lazy criteria/sub-criteria, thereby laying the groundwork for delineating strategies augmenting the suitability of each algorithm. The Simple Additive Weighting (SAW) method can be also applied to order the alternatives; however, its results are not always realistic and are only limited to maximizing criteria, which precludes its application in this context [48,49]. Similarly, the Analytic Hierarchy Process (AHP) can support the evaluation of alternatives but a major concern is the high number of comparisons that experts may undertake due to a large number of alternatives. Moreover, the availability of imprecise data, often encountered in the real world, hinders the potential use of Data Envelopment Analysis (DEA) for underpinning the ranking task [50].

To the best of our knowledge, there are no approaches that integrate the IF-DEMATEL and TOPSIS methods in this context. However, there are approaches that integrate IF-DEMATEL and TOPSIS, with some variations, that have been used for other applications such as performance evaluation of G20 economies (DEMATEL and TOPSIS) [51], research and development project selection (IF-DEMATEL and IF-TOPSIS) [52], estimation of participants in knowledge-intensive crowdsourcing (fuzzy DEMATEL and TOPSIS) [53], hotel information system selection (IVIF-DEMATEL and IVIF-TOPSIS) [54], and risk assessment of hydrogen generation (DEMATEL and TOPSIS) [55]. Accordingly, this paper investigates the novel integration of the IF-DEMATEL and TOPSIS methods as an alternative approach to select the best classification algorithm to support ATS adoption by PwD. This paper is a continuation of the work presented in Ortiz-Barrios et al. [3] and in Ortiz-Barrios et al. [29] to research the use of MCDM techniques that support classifier selection in the context of ATS adoption by PwD.

## 3. Proposed Methodology

**Figure 1:**
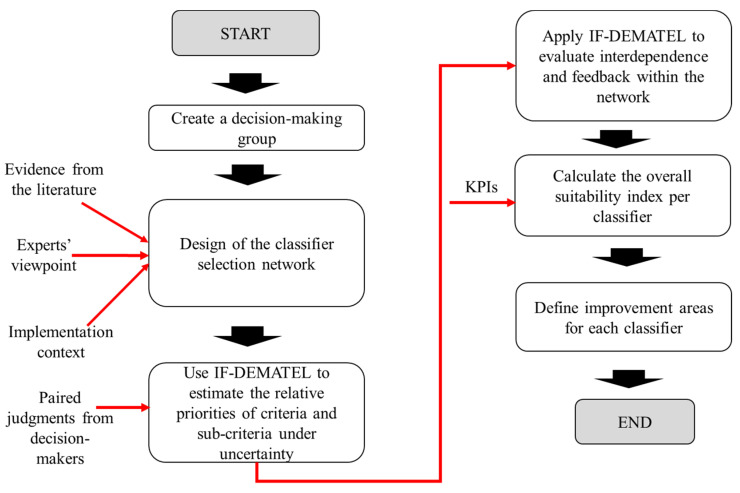
Proposed methodology for selecting the most suitable classifier considering the implementation context of technology adoption.

A six-step approach based on the IF-DEMATEL and TOPSIS methods is proposed for coping with the classifier selection problem in assistive technology adoption, in addition to detecting significant areas for improvement in each alternative algorithm (Figure 1). The general procedure is described as follows:

Step 1. Creation of an expert decision-making group: In this context, it is necessary to rely on pertinent experts providing in-depth insights into the decision network. In particular, it is expected that the participants help in the identification of classifier selection criteria and sub-criteria, the estimation of relative priorities, and the assessment of interdependence and feedback within the network.

Step 2. Design of the classifier selection network: The classifier selection problem has proven to be of a multi-criteria nature. In this respect, criteria and sub-criteria need to be defined taking into consideration the evidence from the literature, experts’ viewpoint, and implementation context.

Step 3. Estimating the relative priorities of criteria and sub-criteria under uncertainty: Once decision elements have been identified, paired comparisons must be made supporting the calculation of relative priorities under the IF-DEMATEL method. The results derived from this application are the backbone for the definition of improvement areas whose intervention can upgrade the classifier development process.

Step 4. Interdependence and feedback evaluation: The second aim pursued through the IF-DEMATEL implementation is the characterization of potential cause–effect relationships within the network. IFS are incorporated to deal with the inherent indeterminacy and uncertainty denoted in human judgments. The outputs resulting from this step can be used for identifying the main drivers behind the effective design, development, and implementation of classification algorithms in the healthcare context.

Step 5. Calculating the overall suitability index per classifier: TOPSIS is employed for determining a suitability index for each candidate algorithm. The classifiers are then decreasingly ranked considering this indicator. The alternative with the highest score is selected for supporting assistive technology adoption in the real world.

Step 6. Definition of improvement areas: Classification developers direct their attention towards overcoming barriers hindering the applicability of algorithms in the practical healthcare scenario. Therefore, identifying these barriers is of great importance for improving the design process. Analyzing the Euclidean distances provided by the TOPSIS application can lay the groundwork for addressing this task.

### 3.1. Intuitionistic Fuzzy Decision Making Trial and Evaluation Laboratory (IF-DEMATEL)

In this section, an initial outline is provided to aid comprehension of the theory supporting the Intuitionistic Fuzzy Sets (IFS). Then, the IF-DEMATEL procedure is thoroughly described. IFS theories were initially introduced by Atanassov [56] and have been widely implemented by practitioners in uncertain decision-making scenarios. These sets are characteristic functions defining the membership, non-membership, and hesitation (unknown information). In IFS, the degrees of validity (membership) and non-validity (non-membership) are equivalent to 1. The IFS fundamental definitions are explained below to establish the mathematical basis underpinning the IF-DEMATEL method.

**Definition** **1:** *Let X be a fixed domain of discourse. Then, an IFS can be denoted as exposed in Equation (1) [57,58]:*

(1) $$I=\{\langle x,\;I(\mu_{I}(x),v_{I}(x))\rangle |x\in X\}$$

*Here* $\mu_{I}(x):X\mapsto [0,1]$ *denotes the degree of membership while* $v_{I}(x):X\mapsto [0,1]$ *represents the degree of non-membership considering the following condition (Equation (2)):*

(2) $$0\leq \mu_{I}(x)+v_{I}(x)\leq 1$$

*The hesitancy degree* $\pi_{I}\left(x\right)$ *is defined as stated in Equation (3):*

(3) $$\pi_{I}(x)=1-\mu_{I}(x)-v_{I}(x),\;x\in X$$

**Definition** **2:** *IFS defuzzification can be performed using Equations (4) and (5). This methodology comprises of two phases described as follows: (1) conversion of IFS into fuzzy classical subsets (Equations (4) and (5)) and (2) implementation of a defuzzification function* f: μ(x)→R*considering a Center of Gravity (COG) method.*

(4) $$C_{\varphi }\left(I\right)=\left\{\langle x,\mu_{I}\left(x\right)+\varphi \pi_{I}\left(x\right),v_{I}\left(x\right)+\left(1-\varphi \right)\pi_{I}\left(x\right)\rangle ,x\epsilon X\right\}\;\mathrm{with}\;\varphi \epsilon \left[0,1\right]$$

$C_{\varphi }\left(I\right)$ is the defuzzificatin operator and represents a classical fuzzy subset with a validity function as expressed in Equation (5) [59]:

(5) $$\mu_{\varphi }\left(x\right)=\mu_{I}\left(x\right)+\varphi \pi_{I}\left(x\right)$$

In this case, φ=0.5 is adopted as a solution for$\;d\left(C_{\varphi }\left(I\right),\;I\right)$ where *d* symbolizes the Euclidean separation. The membership function defining the resulting fuzzy set $C_{0.5}\left(I\right)$ is $\mu \left(x\right)=\frac{1}{2}\left(1+\mu_{I}\left(x\right)-v_{I}\left(x\right)\right)$.

Once the IFS have been introduced, we describe the step-by-step procedure of the IF-DEMATEL method as follows:

Step 1—Identification of decision criteria and sub-criteria related to the classifier selection problem.

Step 2—Definition of the initial direct relation matrix in IFS: The following judgment scale (in IFS) was defined as: Null influence <0.1, 0.9>, Low influence <0.35, 0.6>, Medium influence <0.5, 0.45>, High influence <0.75, 0.2>, and Very high influence <0.9, 0.1>. As evidenced, each judgment is denoted as a 2-tuple intuitionistic style <$\mu_{I}\left(x\right)$, $v_{I}\left(x\right)$> whose hesitation degree $\pi_{I}\left(x\right)$ is calculated using Equation (3). The experts are asked to select one of the scale options when comparing criteria or sub-criteria.

Step 3—Computation of membership functions: In this step, IFS measures are defuzzified considering the algorithm presented by Anzilli and Facchinetti [58] (see Definition (2)). The IFS is first transformed into a classical fuzzy subset by implementing$\;\mu \left(x\right)=\frac{1}{2}\left(1+\mu_{I}\left(x\right)-v_{I}\left(x\right)\right)$. Following this, the calculated subsets are converted into crisp values by first designating a fuzzy triangular number and then using Equations (6) and (7). A matrix $Z_{k}={\left[z_{ij}^{k}\right]}_{nxn}$ with the resulting crisp numbers is derived accordingly considering *k* (*k = 1, 2, …, l)* as the *kth* decision-maker participating in the expert team.

(6) $$\mu \left(\tilde{x}\right)=\left(\tilde{x}-l\right)/\left(m-l\right)$$

(7) $$\tilde{x}=l+\mu \left(\tilde{x}\right)\left(m-l\right)$$

It is also necessary to measure the reliability of IF-DEMATEL results to avoid the inclusion of potential bias from the experts. Most DEMATEL-related applications, however, ignore consistency checking and assume that this information is trustworthy. In this regard, some studies [60] have proposed the use of the corrected item-total correlation as an attempt to deal with this weakness. To increase the evidence base related to this topic, our approach proposes the use of the convergence index, which was taken into consideration when evaluating the internal consistency among experts’ preferences (in this case, the pairwise comparisons in IF-DEMATEL).

Step 4—Aggregation of initial direct-relation matrixes: The $Z_{k}$ matrixes are later aggregated to obtain the matrix $Z={\left[z_{ij}\right]}_{nxn}$ via implementing Equation (8).

(8) $$z_{ij}=\frac{1}{l}\overset{l}{\underset{k=1}{\sum }}z_{ij}^{k},\;\;\;\;\;\;i,j=1,2,\ldots ,n$$

Step 5—Computation of the convergence index: The convergence index, calculated using Equation (9), determines whether the decision makers were accurate when performing the judgments [61]. In Equation (9), *n* denotes the number of experts, and $g_{c}^{ijp}$ and $g_{c}^{ij\left(p-1\right)}$ represent the integration matrix of *n* and *n* − 1, experts respectively. If this index is less than 0.05, the convergence is satisfactory and the experts are therefore considered consistent in their preferences [61].

(9) $$\frac{1}{n\left(n-1\right)}\overset{n}{\underset{i=1}{\sum }}\overset{n}{\underset{j=1}{\sum }}\frac{\left|g_{c}^{ijp}-g_{c}^{ij\left(p-1\right)}\right|}{g_{c}^{ijp}}$$

Step 6—Calculation of the normalized direct-relation matrix (*X*): The initial direct-relation matrix in crisp values is then normalized by applying Equations (10) and (11). Here, *s* denotes the norm used to obtain *X*.

(10) $$X=s^{-1}Z$$

(11) $$s=max\left(\overset{n}{\underset{j=1}{\sum }}z_{ij}\;,\overset{n}{\underset{i=1}{\sum }}z_{ij}\;\right)$$

Step 7—Derivation of the total-influence matrix (*T*): This matrix is computed by adding the direct and indirect effects as expressed in Equation (12).

(12) $$T=X{\left(I-X\right)}^{-1}$$

In Equation (12), *I* represents the identity matrix. After generating *T*, the aforementioned effects are calculated. In particular, *r_i_* is the sum of direct and indirect influences from a particular criterion/sub-criterion to the others, and *d_j_* represents the sum of direct and indirect effects that a decision element receives from the rest. Then, prominence $\left(D+R^{T}\right)$ and relation$\;\left(D-R^{T}\right)$ indicators are computed applying Equations (13) and (14). Specifically, prominence depicts the strength of effects that are dispatched and received by a particular criterion/sub-criterion, whereas relation evidences the net influence that a decision element contributes to the system [6]. If relation is negative, the criterion/sub-criterion is classified as receiver; if it is positive, the decision element is categorized as dispatching.

(13) $$D={\left[d_{i}\right]}_{nx1}={\left(\overset{n}{\underset{j=1}{\sum }}t_{ij}\right)}_{nx1}={\left(t_{i}\right)}_{nx1}$$

(14) $$R={\left[r_{j}\right]}_{1xn}={\left(\overset{n}{\underset{i=1}{\sum }}t_{ij}\right)}_{1xn}={\left(t_{j}\right)}_{1xn}$$

Step 8—Estimate the relative weights of criteria and sub-criteria by implementing Equation (15).

(15) $$w_{i}=\frac{D_{i}+R_{i}}{\sum_{i=1}^{n}D_{i}+R_{i}},\;\;\;\;\;\;\;\;i=1,\;2,\;\ldots \;,\;n$$

Step 9—Draw an Impact Relation Map (IRM) by graphing the dataset (*D* + *R*, *D* − *R*).

### 3.2. Technique for Order of Preference by Similarity to Ideal Solution (TOPSIS)

TOPSIS is an outranking multi-criteria decision-making approach that helps to select the best candidate in consideration of a set of predefined criteria. This method simultaneously measures the distance of each alternative to the best ideal solution and the negative ideal scenario [12,47]. TOPSIS provides a closeness coefficient denoting the suitability of a particular option is so that experts can easily identify the best pathway, and undertake before-and-after performance analysis in a process improvement context. TOPSIS also works with quantitative and commensurable indicators and is therefore adaptable to different indicator systems [7]. Despite this, the rank reversal problem is a limitation for future changes regarding the inclusion of new alternatives and decision criteria. The new version proposed by García-Cascales and Lamata [62] is adopted in this paper to tackle this shortcoming. The modified TOPSIS algorithm is outlined as follows:

- Construct the performance matrix P by considering c classifiers and n decision elements. Each $p_{ij}$ denotes the value of the decision element $D_{j}\;\left(j=1,2,3,\ldots ,n\right)$ in each classifier$\;C_{i}\left(i=1,2,\ldots ,c\right)$.

- Obtain the normalized performance matrix N using Equation (16) where $m_{ij}$ is the modified TOPSIS norm suggested by García-Cascales and Lamata [62]. Estimate $m_{ij}$ via utilizing Equation (17). $n_{ij}$ symbolizes the element of the normalized performance matrix N corresponding to the decision criterion j of each classifier i.
  (16) $$N=P\cdot m_{ij}$$
  (17) $$m_{ij}=\frac{p_{ij}}{p_{ij}\;}$$

- Compute the weighted normalized performance matrix (V) via implementing Equation (18). The sub-criteria priorities $\left(w_{j}\right)$ are given by the IF-DEMATEL technique.
  (18) $$V=\left[w_{j}p_{ij}\right]=\left[v_{ij}\right]$$

- Establish absolute positive $\left(A^{+}\right)$ and negative $\left(A^{-}\right)$ ideal solutions according to García-Cascales and Lamata [62].

- Obtain the Euclidean distance from the positive (PIS) and negative (NIS) ideal solutions for each classifier via utilizing Equations (19) and (20) correspondingly.

Separation from PIS-$S_{i}^{+}$:

(19) $$S_{i}^{+}=\sqrt{\overset{n}{\underset{j=1}{\sum }}{\left(v_{ij}-v_{j}^{+}\right)}^{2}}\;\;\;\;\;\;\;\;i=1,2,\ldots ,c\;$$

Separation from NIS-$S_{i}^{-}$:

(20) $$S_{i}^{-}=\sqrt{\overset{n}{\underset{j=1}{\sum }}{\left(v_{ij}-v_{j}^{-}\right)}^{2}}\;\;\;\;\;\;\;\;i=1,2,\ldots ,c\;$$

- Estimate the relative closeness coefficient to the ideal solution $R_{i}$ (that is, the “suitability index”) of each classifier via Equation (21).
  (21) $$R_{i}=\frac{S_{i}^{-}}{\left(S_{i}^{+}+S_{i}^{-}\right)},\;\;\;\;\;\;0<R_{i}<1,\;\;\;\;\;\;i=1,2,\ldots ,\;c\;$$
  $$R_{i}=1\;if\;A_{i}=A^{+}$$

- Rank the classifiers in a decreasing order based on $R_{i}$ values.

## 4. A Case Study of a Mobile-Based Reminding Solution

Reminding solutions have been found to be promising for improving the quality of life experienced by PwD and their carers. Specifically, they are expected to help PwD to live more independently by compensating for cognitive impairments. Some conditions, however, can limit the potential benefits of these technologies given the inability to adapt the solution to all PwD profiles. It is then of paramount relevance to define if a person can adopt the reminder solution so that negative psychological effects and excess costs can be avoided in the real-world settings. The solution is a video-streaming mobile technology supporting PwD self-management when performing the Activities of Daily Living (ADLs). In particular, this system provides PwD with video reminders recorded by the carers indicating the ADLs that need to be undertaken at certain times. To effectively deploy this application in the healthcare scenario, diverse classifiers have been used by the developers: AdaBoosting (AB), Classification and Regression Tree (CART), Decision Tree (DT), K-Nearest Neighbors (KNN), Naïve Bayes (NB), Neural Network (NN), and Support Vector Machine (SVM). Our approach will aim to select the most appropriate classification algorithms to support the adoption of the mobile-based solution based on the different criteria available, including those from the implementation domain. This section presents the results of the step-by-step procedure followed to pursue this objective.

### 4.1. The Decision-Making Group

The aim of the creation of a decision-making group is three-fold: (i) to define the criteria and sub-criteria that will be considered in the classifier selection problem, (ii) to perform the paired comparisons required by IF-DEMATEL to estimate the weights of criteria and sub-criteria under uncertainty while appraising interdependence, and (iii) to identify the classifier alternatives potentially supporting the discrimination between adopters and non-adopters of the mobile-based solution. The decision-making procedure was guided by a researcher co-authoring this manuscript. Seven professionals with expertise in classification algorithms and involvement in the EU-funded *REMIND* Project were asked to be part of the decision-making group. The focus of *REMIND* is directed towards the creation of more appropriate and efficacious reminding solutions based on user center design, behavioral science, and computational techniques. A brief outline of the decision-makers profile is presented below (Table 1).

The head researcher designed the network model underpinning the classifier selection problem based on the criteria, sub-criteria, and classifier alternatives emanating from the participants, the pertinent literature, and the healthcare context. Furthermore, he instructed the experts on how to correctly perform the pairwise judgments utilizing the IF-DEMATEL scale. The comparisons were completed after a 2-h session through an online data-gathering instrument. The participants also provided the pertinent feedback on the model and the results obtained after the application. The activities here described helped stakeholders to understand the role of a classifier beyond the performance domain and thereby facilitating the alignment of the development process with the real multifactorial context affecting the ATS implementation.

### 4.2. The Classifier Selection Network

**Figure 2:**
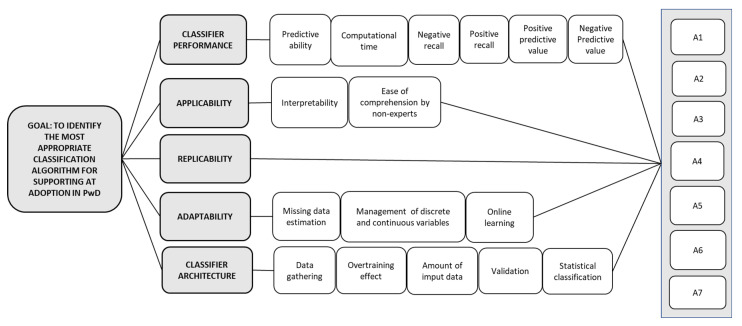
The proposed decision-making structure for selecting the most suitable classifier for AT adoption.

The classifier selection network was discussed with decision makers in two online meetings having a duration of 1 h each, in which it was checked that the classifier selection network was suitable, rational, and comprehensible. The final version of the network model (Figure 2) is composed of five categories, 16 sub-categories, and seven classifier alternatives. The decision elements were extracted from the participants’ point of view, the related reported literature, and the implementation context. The resulting criteria and sub-criteria are applicable to the multitude of neurodegenerative diseases related to dementia. An outline of each category is depicted below (Table 2) followed by a detailed explanation of the sub-categories.

In particular, Predictive Ability (SF1), denoted as the ratio between the number of correct predictions over the total number of predictions, was concluded to be one of the most popular measures utilized in machine learning applications when assessing classifier performance. In a similar vein, *Computational time (SF2)* exhibits the agility of algorithms to estimate and display the predictions (adopter or non-adopter). The idea is to count on algorithms accelerating the discrimination process in the real scenario. Another aspect to be deemed in the *classifier performance* field is *Negative Recall (SF3)*, which symbolizes the proportion of negative class predictions that were effectively discriminated by a particular algorithm from a group of negative cases. Classifiers with large negative recall values are highly preferred because these can help identify non-adopters, which minimizes the intricate psychological impact in both PwD and carers. In parallel, *Positive Recall (SF4)* evidences the proportion of positive classes that are correctly distinguished by a classifier considering a group of positive instances. Algorithms presenting high *positive recall* scores are prioritized for pinpointing prospective PwD candidates that may gain benefits from the proposed ATs. Other sub-criteria within the *Classifier performance* criteria are *Positive predictive value (SF5)* and *Negative predictive value (SC6)*, which represent the rate of negative cases that are accurately categorized by the algorithm.

Another criterion to be assessed in this selection model is the *Applicability (F2)* of the classifier. Two aspects were considered within this domain. First, it is necessary to define if the algorithm is easy to comprehend by the clinicians *(SF7)* so that usage errors can be avoided in the practical context. In this regard, the user is expected to fully know the procedure (i.e., input data collection, classifier structure) so that an agile application of the technology adoption model can be fully undertaken. Similarly, it is important to evaluate the interpretability of the algorithm, which is related to the box type of the classifier. Black-box models are difficult to explain as they do not specify how input data are combined to make predictions; on the contrary, white-box algorithms clearly establish how they function, which significant variables are taken into account, and how predictions are derived. From the implementation perspective, white-box models are preferred to black-box algorithms.

From the *Adaptability (F3)* point of view, it is important to highlight the need for missing data estimation *(SF9)* because healthcare databases are often characterized by the incomplete processing of online formats, which may be a hindrance for the effective training of technology adoption classifiers. In this respect, algorithms with methodological approaches overcoming this barrier are highly suggested. In addition, classifiers are expected to manage variables of different nature (i.e., discrete and continuous) *(SF10)* that may influence the likelihood of adopting a particular AT. Not effectively handling diverse data types may decrease the discrimination ability of the algorithm and thereby minimize its adaptability to the real healthcare context. Finally, the *Online learning (SF11)* sub-criterion evaluates whether the technology adoption model may evolve via incorporating new parameters representing the dynamic healthcare context and the presence of interrelations influencing the uptake of reminding solutions.

The classifier architecture is also considered when selecting the algorithm discriminating technology adoption in PwD. A related aspect is *Data gathering (SF12)*, which verifies whether the feature set of the model can be collated through available data sources and/or simple self-administered surveys. It is also relevant to measure potential overtraining effects *(SF13)* in the model. This pattern is observed when the algorithm performance decreases in learning parameters; that is, the model describes noise or errors instead of real significant relationships between the predictors and response variables. In this regard, classifiers with high overtraining effects cannot feasibly be deployed in the real world. The *amount of input data (SF14)* required by the algorithm to make the predictions is another relevant aspect to consider in the classifier selection model. The number of predictors is directly related to the data registration time, which is a component of the total patient flow time. In consequence, classifiers with a small number of inputs are preferred for real applications. Similarly, it is necessary to use testing data so that we can ensure the generalization ability of the trained algorithm *(SF15)* [63]. Ultimately, *SF16* checks if the classifier is of statistical nature, an aspect of wide benefit in the real healthcare context given its capability of handling vague or unknown values, in addition to presenting a dynamic structure that is adaptable to the technology adoption scenario.

### 4.3. Calculation of Fuzzy Relative Priorities and Interdependence Evaluation: The IF-DEMATEL Approach

The expert group was also required to estimate the relative weights of decision elements and assess the interrelations within the classifier selection model. The decision-makers utilized the judgment scale outlined in Section 3.1, which comprises a group of 2-tuple numbers $<\mu_{I}\left(x\right),\;v_{I}\left(x\right)>$ representing the disagreement, agreement, and neutrality that may emerge during the comparison process. Ongoing support was provided by the project managers to elude inconsistencies and diminish the risk of bias. Table A1 shows the initial direct-relation matrix (in IFSs) resulting from Decision-maker 1 regarding the *Adaptability* sub-elements. Following this, the IFS scores were defuzzified via a two-stage approach. In this regard, the IFSs were converted into their corresponding standard fuzzy subsets by implementing the expression $\mu \left(x\right)=\frac{1}{2}\left(1+\mu_{I}\left(x\right)-v_{I}\left(x\right)\right)$ (see Table A2). A defuzzification function was then implemented to transform the fuzzy subset into a crisp score. In this process, a crisp direct-relation matrix (see example in Table A3 for *Adaptability* cluster) was derived when assigning the scores in Table A2 to the triangular fuzzy number <0, 4, 4>. Additionally, we aggregated the crisp scores of all decision makers by employing the arithmetic average (Table A4). After this, the normalized direct-relation matrix (*X*) (Table A5) was estimated by using Equations (9) and (10). The total influence matrix (Table A6) was then achieved via applying Equation (11). In this case, the standardized Cronbach’s alpha (0.848) was found to be higher than 0.7. Additionally, the convergence index for each matrix were as follows: Criteria (0.013), Classifier performance (0.012), Applicability (0.001), Adaptability (0.002), and Classifier architecture (0.009). Note that inconsistencies introduced by the experts were very low given the non-significant convergences (<0.05). Therefore, their preferences could be used for criteria weighting and interdependence evaluation processes in the decision-making model.

**Figure 3:**
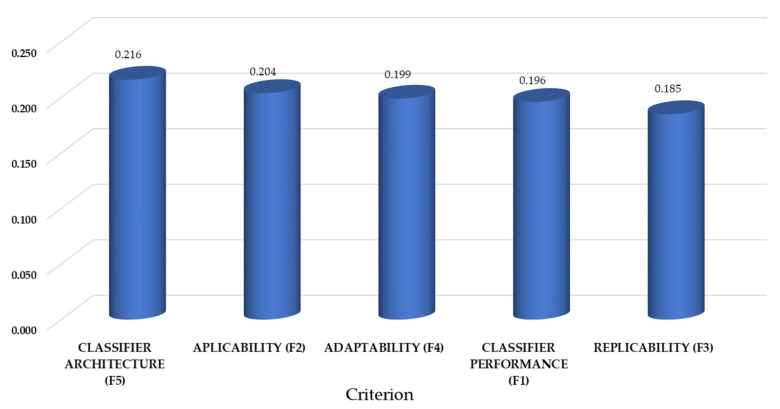
Ranking of classifier selection criteria.

The *D + R^T^* (Equation (12)) and *D – R^T^* (Equation (13)) scores were later derived based upon the total-influence matrixes (Table 3). The criteria and sub-criteria weights were subsequently computed via applying Equation (14). The IF-DEMATEL outcomes uncovered that the most relevant criterion in the selection of classifiers supporting technology adoption for PwD is “Classifier architecture” (F5) with 0.216 (Figure 3). There is, however, a very small difference (0.031) between this factor and the last in the ranking (*Replicability;* GW = 0.185), which denotes the need for upgrading the profile of classifiers considering a multi-criteria perspective. In this regard, developers should not be only skewed to performance measures, as often found in the literature [21,24], but also the intricate aspects of implementation in the real technology adoption context. It is necessary to create classifiers that can accelerate the discrimination of potential adopters and non-adopters during a doctors’ appointment while surpassing the financial barriers limiting their ample use in the public hospital sector. These efforts should be deployed in view of the potential abandonment of these technologies and the increasing burden placed by dementia disease on the existing healthcare systems. By covering all these criteria in the classifier design and development processes, it will be possible to elude negative impacts due to failure, while increasing the likelihood to effectively use these classifiers in the wild.

Table 4 shows the global and local weights of classifier selection sub-criteria. Note that *Ease of comprehension (SF7)* (GW = 0.102) and *Interpretability (SF8)* (GW = 0.102) are the sub-criteria with major contributions to the classifier selection decision. In light of these results, it is evident how classifier performance is not the only aspect to take into consideration when choosing the most convenient algorithm. As clinicians and physicians are not experts in the use of these classifiers, it is then fundamental to previously disseminate how they work, the procedure that users need to follow for obtaining the response variable (adoption or non-adoption), and how the output must be interpreted. In consequence, the learning curve is smoothed and the healthcare process can adopt the algorithm inclusion without significantly extending the appointment time. Similarly, it is recommended to employ white-box classifiers because they are often able to be illustrated and may contain expert knowledge, which is not possible in black-box algorithms.

**Figure 4:**
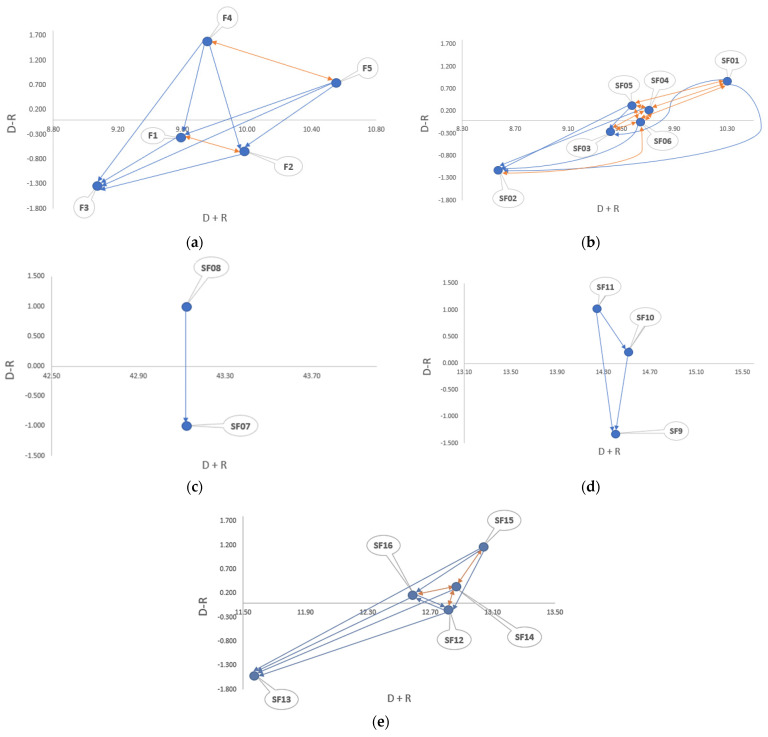
Impact-digraph map for (**a**) criteria, (**b**) classifier performance, (**c**) applicability, (**d**) adaptability, and (**e**) classifier architecture.

By comparison, *Classifier performance (F1)*, *Applicability (F2)*, and *Replicability (F3)* were distinguished as key receivers, and *Adaptability (F4)* and *Classifier architecture (F5)* were found to be the dispatcher elements. It is also important to note that *Classifier architecture (F5)* evidences the highest positive prominence value (10.554) and can be therefore deemed as the main generator of effects in the classifier selection model. In consequence, this factor should be prioritized by developers to facilitate the implementation of the algorithm in the clinical scenario. The approach to be followed during the classifier design greatly affects the remainder of the criteria, as outlined in Figure 4a. For example, not having a sufficient sample size incorporates potential bias or noise in the trained algorithm, which results in low replicability and performance [64,65]. In response, efficient resampling schemes for classifiers can be proposed for addressing the limited sample size problem and upgrading their generalizability accordingly, which is an important aspect in technology adoption, as shown in several investigations [21,66]. Generalizability problems are one of the main barriers to the effective implementation of classifiers in the clinical context. From Figure 4a, we can also infer the presence of feedback interrelations with *Adaptability (F4)*. This is explained by the current dynamics imposed by the dementia disease and the constant pressure experienced by healthcare systems, which demand flexible classifier architectures that can be easily adopted by both public and private hospital sectors. There is then an urgent need for rapidly designing classifiers that reply to the changing context and hence entailing more agile development processes. Equally important is the direct relation (blue arrows) that *Classifier architecture (F5)* exerts on *Applicability (F2)*. Algorithms that can be easily explained and deployed via friendly dynamic visualizations help to flatten the learning curve and diminish potential errors/delays during the discrimination process.

In addition, an influence map was constructed to represent the existing interrelations among *Classifier performance* sub-factors (Figure 4b). In this group, the threshold was assumed to be$\;p=\frac{28.627}{6^{2}}=0.795$. The outcomes uncovered that *Predictive ability (SF1)*, *Positive recall (SF4)*, and *Positive predictive value (SF5)* are the dispatchers, whereas *Computational time (SF2)*, *Negative recall (SF3)*, and *Negative predictive value (SF6)* are the receivers. Moreover, various bi-directional relations (orange arrows) are clear from the graph: SF01-SF05, SF01-SF04, SF01-SF06, SF03-SF06, SF05-SF06, SF04-SF05, SF02-SF06, and SF03-SF06. These results are in agreement with the conclusions presented in Zhou and Liu [67] and Pereira et al. [68], which also indicated interdependence among different classifier performance measures. Aligned with the above, *Computational time (SF2)* (also called “run time”) has been found to be highly correlated with *Predictive ability (SF1)*. In practice, some developers may sacrifice accuracy to obtain lower run times. The challenge is how to obtain the best algorithm performance considering both measures. A related effort in this research line is registered by Doan and Kalita [69], who proposed the *Multivariate Adaptive Regression Splines (MARS)* approach for predicting the computational time of machine learning models considering the cost of training and accuracy. Despite this, more studies are required to develop high-accuracy classifiers that can be employed in the healthcare technology adoption context while requiring short computational times. This would be an important contribution considering that the algorithm’s run time will affect the appointment time and, therefore, the patient flows within the hospital.

The prominence-relation set was also derived for evaluating the interdependence among the *Applicability* sub-factors (Figure 4c). The reference score established for this cluster was $p=\frac{43.118}{2^{2}}=10.779$. The map configuration reveals that *Ease of comprehension (SF7)* is the receiver and *Interpretability (SF8)* is the dispatcher. A staggering aspect is the high *D+R^T^* observed in this cluster (43.118). All these findings are underpinned by the fact that interpretable classifiers make informed decisions and can be therefore fundamental in facilitating the discrimination between adopters and non-adopters, in addition to identifying the reasons supporting this decision. The greater the interpretability of a classifier, the easier for the clinicians and physicians to comprehend why a particular PwD may adopt a particular AT. As stated by Doshi-Velez and Kim [70], the need for interpretability emerges from an incomplete view of the implementation context, which is related to the creation of classifiers partially responding to the technology adoption problem. In view of the above, more interpretable classifiers should be designed in line with the healthcare real-world requirements to bridge the current gap observed between theory and practice.

There are also existing interrelations among *Adaptability* sub-criteria (Figure 4d). In particular, the accepted threshold for this cluster was set as$\;p=\frac{21.596}{3^{2}}=2.40$. Unsurprisingly, the digraph uncovers that *Missing data estimation (SF9)* is the only receiver, whereas *Management of continuous and discrete variables (SF10)* and *Online learning (SF11)* are the dispatchers. It is well known that missing data approaches may differ depending on the variables’ nature, as outlined in Jakobsen et al. [71]. In fact, imputation methods are differentiated considering whether the variable is categorical or continuous. Online learning, in contrast, is feasible as missing data related to significant features can be properly managed. Not addressing this problem may cause loss of important data, the introduction of bias, and the consequent reduction in potential statistical power. Data scientists and classifier developers are then advised to focus on the aforementioned aspects to upgrade the adaptability of the algorithms to the complex and dynamic nature of the healthcare technology adoption context.

An influential-relation graph was also elaborated to analyze interdependencies and feedback within the *Classifier architecture* cluster (Figure 4e). In this case, the accepted threshold value was defined as $p=\frac{31.453}{5^{2}}=1.258$**.** The map clearly evidences that *Data gathering (SF12)* and *Overtraining effect (SF13)* are part of the effect group, whereas *Amount of input data (SF14), Validation (SF15),* and *Statistical classification (SF16)* are included in the cause cluster. On the other side, bi-directional relations are noted in SF12-SF14, SF14-SF15, and SF14-SF16, which reinforces the importance of the *Amount of input data (SF14)* within the cluster. In this respect, it is clear that the data collection process will be more complex and time consuming as the number of input data required by the classifier increases. Furthermore, the addition of non-significant or irrelevant features may augment the overtraining effect observed in the model and may cause certain performance deterioration during validation. This is of particular interest considering that an overfitted classifier will be unable to effectively perform new predictions. Going beyond these results, it is relevant to note that statistical classification may be downgraded if some predictors are not incorporated into the model. The challenge is to correctly identify the characteristics that may increase the statistical power of the model by validating their predictive ability through significance tests.

### 4.4. Calculation of Suitability Index per Classifier and Detection of Improvement Opportunities: The Modified TOPSIS Approach

This section presents the application of the modified TOPSIS approach whose principal objective is two-fold: (i) to estimate the suitability index of seven classification algorithms (A1: Neural Networks, A2: Decision Tree, A3: Support Vector Machine, A4: Naïve Bayes, A5: AdaBoost, A6: Classification and Regression Tree, and A7: k-Nearest Neighbor), and (ii) to identify those characteristics that should be improved in each classifier to upgrade its suitability in assistive technology adoption for PwD. The TOPSIS implementation begins with the definition of a Key Performance Index (KPI) per each sub-factor/factor (Table 5). The mathematical formula used for the calculation of each KPI is also shown in Table 5. The performance matrix P (Table A7) is then derived by incorporating the sub-factor/factor values, the classifier candidates, the global weights estimated via IF-DEMATEL, and the “absolute” positive $\left(A^{+}\right)$/negative $\left(A^{-}\right)$ ideal solutions.

**Figure 5:**
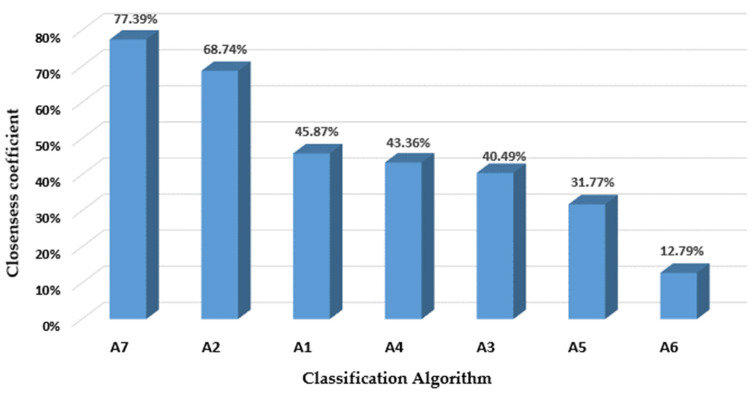
Ranking of alternative classification algorithms considered as a support for the adoption of a mobile-based technology in PwD.

TOPSIS calculations were undertaken considering the seven phases outlined in Section 3.2. The total Euclidean distance of each algorithm (A1, A2, A3, A4, A5, A6, and A7) from the ideal (Si+) (Table A8) and the anti-ideal (Si-) solutions (Table A9) were determined using Equations (19) and (20), respectively. The contribution of each sub-factor/factor to the total distance from the best and worst scenarios is also depicted in Table A8 and Table A9, correspondingly. Ultimately, a suitability index for each algorithm was estimated by applying Equation (21). Figure 5 presents the final TOPSIS measures, i.e., the suitability indexes of classifiers. Upon analyzing the algorithms’ scores, it was clear that A7 (k-Nearest Neighbor;$\;CC_{7}{}^{*}=77.39\%$) was identified as the most suitable classifier for underpinning the adoption of the mobile-based technology in PwD, whereas A2 was ranked in second place (Decision tree;$\;CC_{2}{}^{*}=68.74\%$). Similarly, it can be inferred that only A7 attained a high suitability level (75% ≤ $CC_{i}{}^{*}$ ≤ 100%), whereas A2 demonstrated medium suitability (50% ≤ $CC_{i}{}^{*}$ ≤ 75%), and low suitability (25% ≤ $CC_{i}{}^{*}$ ≤50%) was found in A1, A4, A3, and A5. The least recommended classifier, in this case, is A6, which was found to have very low suitability (0% ≤ $CC_{i}{}^{*}$ ≤25%). The next step will be to identify the weaknesses of each algorithm so developers and data scientists can deploy further improvements, thus increasing the algorithms’ suitability to the real healthcare scenario. For this purpose, it is necessary to discriminate those decision elements whose separation from the ideal solution $v_{j}^{+}$ is significantly over 0 or those whose distance to the anti-ideal scenario $v_{j}^{-}$ is equal to 0. For instance, A4 (Naïve Bayes) evidences the lowest prediction accuracy ($0.349,{\left(V_{14}-V_{1}^{+}\right)}^{2}=0.00108)$; in this regard, it is suggested to incorporate other significant variables that may enhance the potential discrimination between PwD adopters and non-adopters of the mobile-based technology. Another alternative is cross-validation, which entails separating the datasets into groups and then modifying the prediction cohort in each iteration. Moreover, other normalization approaches may be tested for improving the training process of the classifier. By comparison, A6 (Classification and Regression Tree) presents the highest average run time ($5,{\left(V_{26}-V_{2}^{+}\right)}^{2}=0.01346)$, which diminishes the likelihood that it can be implemented in the real clinical scenario. In this respect, it is advised to transform or modify the input variables, in addition to implementing accelerated methods for finite-sum minimization, as outlined in Zhang and Xiao [72], and Lan and Zhou [73]. By comparison, A4 ($0.239,{\left(V_{34}-V_{3}^{-}\right)}^{2}=0)$, A5 ($0.185,{\left(V_{35}-V_{3}^{-}\right)}^{2}=0)$, A6 ($0.371,{\left(V_{36}-V_{3}^{-}\right)}^{2}=0)$, and A7 ($0.212,{\left(V_{37}-V_{3}^{-}\right)}^{2}=0)$ were found to have low average recall (-). Improving this metric may entail augmenting the sample size and/or modifying the probability threshold at which negative classes are discriminated against without significantly compromising other performance measures. The same situation was identified regarding the classification of positive classes in A3 ($0.212,{\left(V_{43}-V_{4}^{-}\right)}^{2}=0)$, A5 ($0.239,{\left(V_{44}-V_{4}^{-}\right)}^{2}=0)$, and A6 ($0.132,{\left(V_{46}-V_{4}^{-}\right)}^{2}=0$, for which the recommendations set in classifiers with low negative recall are also applicable but with a focus on the positive instances. Moreover, low positive predictive values were identified in A3 ($0.239,{\left(V_{53}-V_{5}^{-}\right)}^{2}=0)$, A5 ($0.212,{\left(V_{55}-V_{5}^{-}\right)}^{2}=0)$, A6 ($0.132,{\left(V_{56}-V_{5}^{-}\right)}^{2}=0)$, and A7 ($0.239,{\left(V_{57}-V_{5}^{-}\right)}^{2}=0)$. In this respect, it is suggested to remove irrelevant features from the dataset, in addition to adjusting the hyper-parameters and probability threshold. These strategies can be also replicated in classifiers with small negative predictive values: A4 ($0.159,{\left(V_{64}-V_{6}^{-}\right)}^{2}=0)$, A5 ($0.159,{\left(V_{65}-V_{6}^{-}\right)}^{2}=0)$, and A7 ($0.265,{\left(V_{76}-V_{6}^{-}\right)}^{2}=0)$. An aspect of great importance in this application is to determine how comprehensible the classifier is. The outcomes revealed that A1 $\left(0,{\left(V_{71}-V_{7}^{+}\right)}^{2}=0.01040\right)$, A2 $\left(0,{\left(V_{72}-V_{7}^{+}\right)}^{2}=0.01040\right)$, A3 $\left(0,{\left(V_{73}-V_{7}^{+}\right)}^{2}=0.01040\right)$, and A5 $\left(0,{\left(V_{75}-V_{7}^{+}\right)}^{2}=0.01040\right)$ were complex to understand by clinicians and physicians. Machine learning models are being largely applied in healthcare contexts, and more comprehension on their benefits and applicability is needed by funding agencies and related institutions [74]. From this work, it is evident that poor efforts have been made to address this challenge. In reply, we advise adopting user-friendly interfaces in conjunction with better training, and the use of user-centered approaches prior to the classifier implementation in real-world setting. Similarly, A1$\;\left(0,{\left(V_{81}-V_{8}^{+}\right)}^{2}=0.01040\right)$, A3$\;\left(0,{\left(V_{83}-V_{8}^{+}\right)}^{2}=0.01040\right)$, and A5 $\left(0,{\left(V_{85}-V_{8}^{+}\right)}^{2}=0.01040\right)$ are black-box algorithms. In this respect, efforts should be directed towards characterizing the internal structures of these classifiers; that is, obtaining a comprehensible description of the process producing the prediction. This is important considering the need for enhancing the performance and reducing bias, in addition to increasing the clinicians’ engagement during the implementation process. To cope with this problem, Selvaraju et al. [75] proposed the gradient-weighted class activation maps providing heat graphs that show the inner workings of the neural network algorithm. In addition to the above, A3$\;\left(0,{\left(V_{F33}-V_{F3}^{+}\right)}^{2}=0.03423\right)$, A4 $\left(0,{\left(V_{F34}-V_{F3}^{+}\right)}^{2}=0.03423\right)$, A5 $\left(0,{\left(V_{F35}-V_{F3}^{+}\right)}^{2}=0.03423\right)$, and A6 $\left(0,{\left(V_{F36}-V_{F3}^{+}\right)}^{2}=0.03423\right)$ were found to have a learning process costing over GBP 727.48. Using public clouds may be a strategy for reducing the costs; at present, it is suggested that data scientists employ more optimized processors with less storage and computing power. Another aspect to be considered in this analysis is the lack of missing data estimation, which was detected as a weakness in A3 ($0,{\left(V_{93}-V_{9}^{-}\right)}^{2}=0)$. Several actions may emerge to tackle this hurdle: (i) remove rows having missing values, (ii) apply imputation methods for qualitative or numerical data, and (iii) employ prediction models based on predictors with no nulls. Equally relevant is the management of both discrete and continuous variables, which was identified as a shortcoming in A1 ($0,{\left(V_{101}-V_{10}^{-}\right)}^{2}=0$). In this regard, robust mixed-data models have been proposed in the literature so that these types of variables can be simultaneously considered for the classification [76,77]. Moreover, A5 ($0,{\left(V_{125}-V_{12}^{-}\right)}^{2}=0$) and A7 ($0,{\left(V_{127}-V_{12}^{-}\right)}^{2}=0$) were defined as classifiers whose feature set cannot be collated through available data sources and/or simple self-administered surveys. Data acquisition should be simplified by first establishing the data-driven culture within the healthcare institutions, followed by designing centralized information gathering instruments. In this sense, either data warehouses or data lakes may be useful avenues to address the complexity of data collection. In addition to these proposals, it is necessary to remove any tedious manual recording and bureaucratic data-collection procedures that may be alternatively replaced by robotic process automation systems. Finally, as A1 ($0,{\left(V_{161}-V_{16}^{-}\right)}^{2}=0$) and A2 ($0,{\left(V_{162}-V_{16}^{-}\right)}^{2}=0$) are not based on probabilistic modelling, a useful path may include a combination with statistical approaches so that classification accuracies can be meaningfully improved while fully exploiting the advantages of each method [78]. In a different case study (involving another reminding solution), the developers and decision makers were required to remeasure the KPIs and include the results in the TOPSIS performance matrix so that new ranking and intervention opportunities can be obtained. If the technological solution under study is for another disease, new criteria and sub-criteria may be incorporated into the current classifier selection network for which new comparisons containing these decision elements can be needed. For the latter, it will be also necessary to calculate the priorities and define new KPIs representing the inserted criteria and sub-criteria.

### 4.5. Validation Study: Contrasting TOPSIS Results with VIKOR and SAW

**Figure 6:**
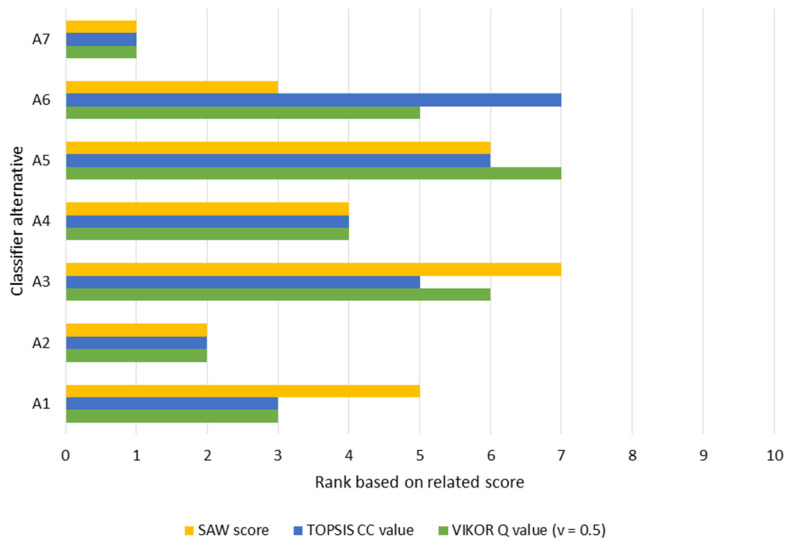
Contrast between SAW, TOPSIS, and VIKOR rankings.

It is fundamental to evaluate the accuracy of the results despite the robust methodological approach proposed within this paper. In this sense, we contrasted TOPSIS with two different outranking methods (VIKOR and SAW). The ranking resulting from each technique is outlined in Figure 6. Based on this graph, there is no variation in the rankings of classifiers A2, A4, and A7. There are some alterations in the rankings of the lasting algorithms due to the internal structuring of each method, although this is not very inconsistent. As highlighted in the reported literature [79], no variation has been seen in ranking the most suitable classifier (A7). Therefore, it was proven that the implementation of the proposed approach in this problem generates reliable results.

**Figure 7:**
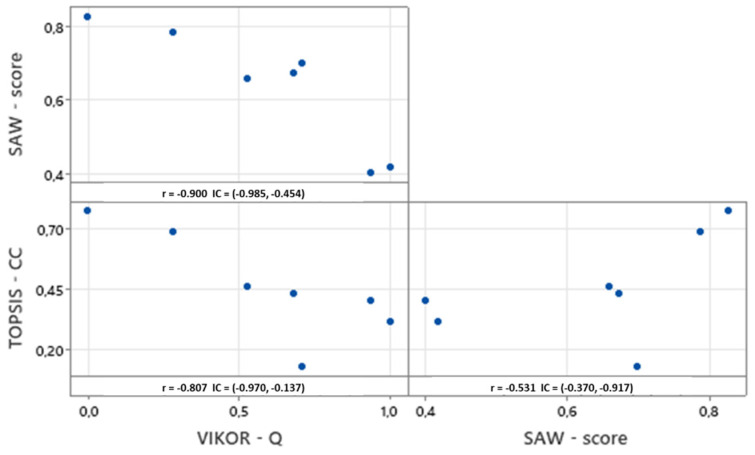
Pearson correlation results (confidence interval = 95.0%).

**Figure 8:**
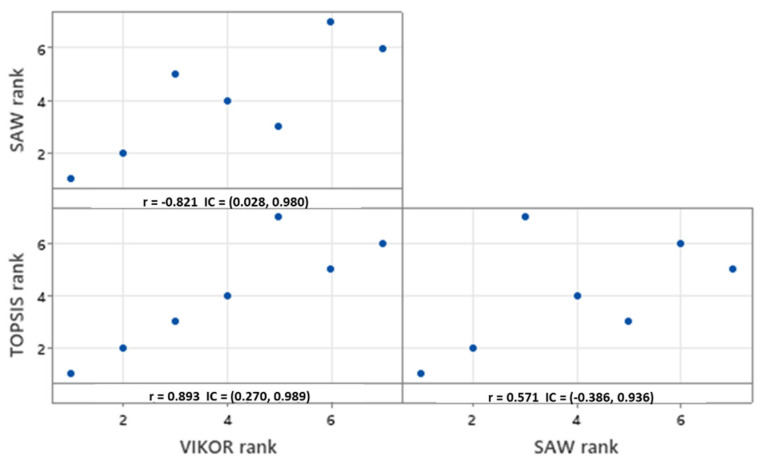
Spearman rank correlation results (confidence interval = 95.0%).

We also validated these outcomes by undertaking the Pearson and Spearman correlation tests for both the final scores of the techniques (*Q* value (v = 0.5) in VIKOR, the closeness coefficient-CC in TOPSIS, and the final score value in SAW) and the resulting rankings. The Pearson correlation test (Figure 7) showed that the final scores for the classifier alternatives are correlated with absolute *r* values ranging from 0.531 to 0.900. Specifically, TOPSIS and VIKOR evidenced the highest correlation (−0.807; 95% CI −0.970, −0.137). It should be mentioned here that *r* is negative because the low *Q* score (closer to zero) in the VIKOR technique denotes that the classifier is more suitable for implementation in real healthcare settings. Similar conclusions were obtained regarding the correlation among ranks. In this case, the Spearman rank correlation metrics were found to vary from 0.571 to 0.893 (Figure 8). Similarly, VIKOR and TOPSIS rankings were found to be highly correlated (0.893; 95% CI 0.270–0.989).

## 5. Concluding Remarks and Future Directions

PwD can improve their quality of life through the support of assistive technologies, enabling them to perform their daily activities in a more natural manner with a greater degree of autonomy in their decisions. Similarly, ATs may provide an opportunity to alleviate the burden experienced by their carers while laying the groundwork for monitoring the disease progress over time. The main barrier, however, is the presence of drivers hampering the wider adoption of these solutions, which results in the need to correctly discriminate between adopters and non-adopters. Although different classifiers have been proposed to address this necessity, the decision about which algorithm should be used goes beyond performance metrics and involves aspects characterizing the healthcare sector and implementation context.

Considering the aforementioned rationale, this paper presents an integrated method based on IF-DEMATEL and modified TOPSIS methods to select the most appropriate classifier underpinning technology adoption in PwD. The proposed knowledge-based approach fully exploits the advantages of these methods and minimizes the gap between theory and practice so that the classifiers can be transferable to a wider number of hospitals currently admitting and treating PwD. The case study outlined here refers to a mobile-based technology whose adoption process can be potentially driven by seven alternative classifiers that were evaluated via a multi-criteria network of five criteria and sixteen sub-criteria.

Three key outcomes were derived from this intervention. The first result was the definition of the most important criterion in classifier selection. The IF-DEMATEL results revealed that the most relevant factor in the selection of algorithms underpinning technology adoption for PwD was “Classifier architecture” (F5), with a value of 0.216. Despite this, only a small difference (0.031) was observed between this criterion and the fifth in the ranking (*Replicability*; GW = 0.185), which evidences the necessity for improving the profile of classifiers via a multi-criteria strategy. By comparison, *Classifier performance (F1)*, *Applicability (F2)*, and *Replicability (F3)* were classified into the effect group, whereas *Adaptability (F4)* and *Classifier architecture (F5)* were concluded to be of dispatching nature. It is also relevant to outline that *Classifier architecture (F5)* was found to be the main generator of effects in the classifier selection model (D + R^T^ = 10.554) and particular attention should be therefore paid to this aspect during the development process. By comparison, TOPSIS showed that KNN, with a closeness coefficient value of 77.39%, followed by DT, with 68.74%, are the most appropriate classifiers for supporting the discrimination process between adopters and non-adopters of the mobile-based technology depicted in this particular case. In this regard, further recommendations were provided to upgrade the eligibility of the candidate classifier for their use in the real healthcare world.

Future research should be directed towards using this approach in other ATs to verify if other considerations may affect their successful implementation in the clinical scenario. In this regard, new criteria related to the financial and medical staff domains may be added to provide a more robust basis for decision making in technology adoption. It is also advised to incorporate interval data in the ranking method to obtain a more realistic approach considering the expected variation of KPIs. Implementing other new generation MCDM methods, including Complex Proportional Assessment (COPRAS), Best and Worst Method (BWM), and other fuzzy approaches based on spherical or neutrosophic sets, is suggested for comparative analysis and validation of the robustness of our results in terms of criteria weighting and ranking of classifiers. Similarly, a mobile application will be developed to calculate the likelihood of technology adoption based on the selected classifier. Thereby, the outcomes of the approach proposed in this paper can be effectively transferred to the PwD and their carers. Moreover, the resulting outputs will be incorporated into the algorithm development process to improve the suitability of low-performance classifiers to the real healthcare context considering the weaknesses detected in this study. Furthermore, our classifier selection approach can be applied to other areas, such as stock index price movement prediction, intruder detection systems, bankruptcy prediction, and chemical fertilizer selection, which evidences the wide range of decision-making scenarios that may benefit from this methodological proposal.

## Figures and Tables

**Table 1:** Profile of decision-making participants.

Expert	Profession	Experience (Years)	Current Position	Participation in the Design of Reminding Technologies
1	Biomedical Engineering	>20	Professor in Biomedical Engineering	Yes
2	Systems Engineering	>10	Lecturer in Data Analytics	Yes
3	Systems Engineering	>20	Senior Lecturer in Ambient Assisted Living	Yes
4	Systems Engineering	>10	Professor of Image Processing	Yes
5	Biomedical Engineering	>10	Assistant Professor in Signal Analysis	Yes
6	Industrial Engineering	>20	Associate Professor	Yes
7	Systems Engineering	>10	Associate Professor	Yes

**Table 2:** Description of classifier selection criteria.

Classifier Selection Criterion	Sub-Criteria	Definition
Classifier performance (F1)	Predictive ability (SF1) Computational time (SF2) Negative recall (SF3) Positive recall (SF4) Positive predictive value (SF5) Negative predictive value (SF6)	It measures the predictive capability of a classification algorithm; in this context, how well the classifier distinguishes adopters and non-adopters of a particular AT [20].
Applicability (F2)	Ease of comprehension by non-experts (SF7) Interpretability (SF8)	This factor denotes how explainable the algorithm is and verifies whether it is easy to understand by clinicians who are often unskilled in this kind of application. This is of interest considering that medical staff will be directly involved in the classifier implementation.
Replicability (F3)	No sub-criteria	This criterion considers the financial investment underpinning the classifier development process as well as its validation in the practical scenario.
Adaptability (F4)	Missing data estimation (SF9) Management of discrete and continuous variables (SF10) Online learning (SF11)	It evaluates how flexible the algorithm is when addressing common data drawbacks (i.e. missing data), different implementation conditions, and diverse variable types. Not effectively responding to this context may limit the application of the classifier in the real world.
Classifier architecture (F5)	Data gathering (SF12) Overtraining effect (SF13) Amount of input data (SF14) Validation (SF15) Statistical classification (SF16)	It exhibits different classifier design aspects including data gleaning, training, and validation which may flatten the learning curve of clinicians while laying the groundwork for the design of agile healthcare processes for PwD.

**Table 3:** Prominence and relation values within the classifier selection model.

Criterion (F)/Sub-Criterion (SF)	*D + R^T^*	*D − R^T^*	Dispatcher	Receiver
**Classifier performance (F1)**	**9.595**	**−0.371**		X
Predictive ability (SF1)	10.306	0.883	X	
Computational time (SF2)	8.582	−1.141		X
Negative recall (SF3)	9.425	−0.289		X
Positive recall (SF4)	9.711	0.230	X	
Positive predictive value (SF5)	9.583	0.376	X	
Negative predictive vaalue (SF6)	9.647	−0.058		X
**Applicability (F2)**	**9.991**	**−0.641**		X
Ease of comprehension (SF7)	43.118	−1.000		X
Interpretability (SF8)	43.118	1.000	X	
**Replicability (F3)**	**9.075**	**−1.333**		X
**Adaptability (F4)**	**9.755**	**1.609**	X	
Missing data estimation (SF9)	14.416	−1.314		X
Management of continuous and discrete variables (SF10)	14.524	0.258	X	
Online learning (SF11)	14.252	1.056	X	
**Classifier architecture (F5)**	**10.554**	**0.736**	X	
Data gathering (SF12)	12.827	−0.173		X
Overtraining effect (SF13)	11.584	−1.520		X
Amount of input data (SF14)	12.868	0.322	X	
Validation (SF15)	13.039	1.174	X	
Statistical classification (SF16)	12.589	0.198	X	

**Table 4:** Local and global weights of classifier selection sub-criteria.

	IF-DEMATEL	
Criterion (F)/Sub-Criterion (SF)	LW	GW
**Classifier performance (F1)**	**0.196**	
Predictive ability (SF1)	0.180	0.035
Computational time (SF2)	0.150	0.029
Negative recall (SF3)	0.165	0.032
Positive recall (SF4)	0.170	0.033
Positive predictive value (SF5)	0.167	0.033
Negative predictive value (SF6)	0.168	0.033
**Applicability (F2)**	**0.204**	
Ease of comprehension (SF7)	0.500	0.102
Interpretability (SF8)	0.500	0.102
**Replicability (F3)**	**0.216**	
**Adaptability (F4)**	**0.199**	
Missing data estimation (SF9)	0.334	0.066
Management of continuous and discrete variables (SF10)	0.336	0.067
Online learning (SF11)	0.330	0.066
**Classifier architecture (F5)**	**0.216**	
Data gathering (SF12)	0.204	0.044
Overtraining effect (SF13)	0.184	0.040
Amount of input data (SF14)	0.205	0.044
Validation (SF15)	0.207	0.045
Statistical classification (SF16)	0.200	0.043

**Table 5:** List of key performance indexes utilized in the modified TOPSIS.

Sub-Factor/Factor	Key Performance İndex	Mathematical Formula
Predictive ability (SF1)	Average accuracy	$\overset{n}{\underset{i=1}{\sum }}\frac{TN+TP}{\left(TP+FP+FN+TN\right)\;*\;n}*100$ TN: True negative predictions TP: True positive predictions FP: False positive predictions FN: False negative predictions n: Number of iterations
Computational time (SF2)	Average run time	$\overset{10}{\underset{i=1}{\sum }}\frac{RT_{i}}{10}$
Negative recall (SF3)	Average recall (-)	$\overset{10}{\underset{i=1}{\sum }}\frac{TN}{\left(TN+FP\right)}*10$ TN: True negative predictions FP: False positive predictions
Positive recall (SF4)	Average recall (+)	$\overset{10}{\underset{i=1}{\sum }}\frac{TP}{\left(TP+FN\right)}*10$ TP: True positive predictions FN: False negative predictions
Positive predictive value (SF5)	Average precision (+)	$\overset{10}{\underset{i=1}{\sum }}\frac{TP}{\left(TP+FP\right)}*10$ TP: True positive predictions FP: False positive predictions
Negative predictive value (SF6)	Average precision (-)	$\overset{10}{\underset{i=1}{\sum }}\frac{TN}{\left(TN+FN\right)}*10$ TN: True negative predictions FN: False negative predictions
Ease of comprehension (SF7)	Model appropriation	If the algorithm is easy to appropriate by physicians and clinicians (1); otherwise (0)
Interpretability (SF8)	Box type	If it a black-box algorithm (0); white-box algorithm (1)
Replicability (F3)	Unit replication cost	It the learning process cost is higher than £727.48 (0); otherwise (1)
Missing data estimation (SF9)	Capability of missing data management	If the algorithm is capable of handling missing data (1); otherwise (0)
Management of continuous and discrete variables (SF10)	Management of continuous and discrete variables	If the algorithm works with both continuous and discrete variables (1); otherwise (0)
Online learning (SF11)	Online learning	If the algorithm is of online-learning type (1); otherwise (0)
Data gathering (SF12)	Easiness of data collation	If the feature set of the model can be collated through available data sources and/or simple self-administered surveys (1); otherwise (0)
Overtraining effect (SF13)	Overtraining	If the algorithm evidences overtraining effect (0); otherwise (1)
Amount of input data (SF14)	Number of input variables	Number of patient features that the classifier needs for displaying the prediction
Validation (SF15)	Access to validation datasets	If the algorithm has access to validated datasets (1); otherwise (0)
Statistical classification (SF16)	Algorithm nature	If the algorithm is based on statistical modelling (1); otherwise (0)

## Data Availability

The data presented in this study are available in the Appendix A within this article.

## Appendix A

**Table A1:** Direct-relation matrix in Intuitionistic Fuzzy Sets—Decision-maker 1 (*Adaptability* sub-criteria).

	SF9		SF10		SF11	
SF9	0	0	0.90	0.10	0.50	0.45
SF10	0.50	0.45	0	0	0.90	0.10
SF11	0.50	0.45	0.10	0.90	0	0

**Table A2:** Direct-relation matrix in standard fuzzy subsets—Decision-maker 1 (*Adaptability* sub-criteria).

	SF9	SF10	SF11
SF9	0	0.90	0.53
SF10	0.53	0	0.90
SF11	0.53	0.10	0

**Table A3:** Crisp direct-relation matrix—Decision-maker 1 (*Adaptability* sub-criteria).

	SF9	SF10	SF11
SF9	0	3.60	2.10
SF10	2.10	0	3.60
SF11	2.10	0.40	0

**Table A4:** Aggregated crisp direct-relation matrix for *Adaptability* matrix.

	SF9	SF10	SF11
SF9	0	2.18	1.91
SF10	2.65	0	2.23
SF11	2.66	2.45	0

**Table A5:** Normalized direct-relation matrix for *Adaptability* sub-criteria.

	SF9	SF10	SF11
SF9	0	0.409	0.360
SF10	0.499	0	0.419
SF11	0.501	0.461	0

**Table A6:** Total influence matrix for *Adaptability* sub-criteria.

	SF9	SF10	SF11	D
SF9	2.184	2.270	2.097	6.551
SF10	2.796	2.234	2.361	7.391
SF11	2.885	2.629	2.140	7.654
R	7.865	7.133	6.598	

**Table A7:** The performance matrix in TOPSIS application.

	SF1	SF2	SF3	SF4	SF5	SF6	SF7	SF8	F3
A1	0.85	1	0.664	0.584	0.717	0.61	0	0	1
A2	0.85	1	0.504	0.637	0.637	0.425	0	1	1
A3	0.825	1	0.478	0.212	0.239	0.557	0	0	0
A4	0.349	1	0.239	0.584	0.504	0.159	1	1	0
A5	0.875	2	0.185	0.239	0.212	0.159	0	0	0
A6	0.825	5	0.371	0.132	0.132	0.504	1	1	0
A7	0.825	1	0.212	0.265	0.239	0.265	1	1	1
A+	1	1	1	1	1	1	1	1	1
A-	0	5	0	0	0	0	0	0	0
W	0.035	0.029	0.032	0.033	0.033	0.033	0.102	0.102	0.185
	SF9	SF10	SF11	SF12	SF13	SF14	SF15	SF16	
A1	1	0	1	1	1	2	1	0	
A2	1	1	1	1	1	1	1	0	
A3	0	1	1	1	0	1	1	1	
A4	1	1	1	1	0	2	1	1	
A5	1	1	1	0	0	2	1	1	
A6	1	1	1	1	0	2	1	1	
A7	1	1	1	0	1	1	1	1	
A+	1	1	1	1	0	1	1	1	
A-	0	0	0	0	1	2	0	0	
W	0.066	0.067	0.066	0.044	0.04	0.044	0.045	0.043	

**Table A8:** Matrix of distances to the ideal solution.

	SF1	SF2	SF3	SF4	SF5	SF6	SF7	SF8	F3
A1	0.00050	0.00021	0.00062	0.00075	0.00060	0.00072	0.01040	0.01040	0.00856
A2	0.00009	0.00000	0.00057	0.00038	0.00038	0.00073	0.01040	0.00000	0.00000
A3	0.00012	0.00000	0.00061	0.00099	0.00097	0.00052	0.01040	0.01040	0.03423
A4	0.00108	0.00021	0.00097	0.00075	0.00083	0.00106	0.00260	0.00260	0.03423
A5	0.00047	0.00084	0.00099	0.00103	0.00104	0.00106	0.01040	0.01040	0.03423
A6	0.00091	0.01346	0.00097	0.00108	0.00108	0.00098	0.00666	0.00666	0.03423
A7	0.00012	0.00000	0.00093	0.00094	0.00097	0.00094	0.00000	0.00000	0.00000
	SF9	SF10	SF11	SF12	SF13	SF14	SF15	SF16	Si+
A1	0.00109	0.00449	0.00109	0.00048	0.00040	0.00194	0.00051	0.00185	0.21121
A2	0.00000	0.00000	0.00000	0.00000	0.00160	0.00000	0.00000	0.00185	0.12656
A3	0.00436	0.00000	0.00000	0.00000	0.00000	0.00000	0.00000	0.00000	0.25021
A4	0.00109	0.00112	0.00109	0.00048	0.00000	0.00194	0.00051	0.00046	0.22586
A5	0.00109	0.00112	0.00109	0.00194	0.00000	0.00194	0.00051	0.00046	0.26192
A6	0.00279	0.00287	0.00279	0.00124	0.00000	0.00008	0.00130	0.00118	0.27977
A7	0.00000	0.00000	0.00000	0.00194	0.00160	0.00000	0.00000	0.00000	0.08629

**Table A9:** Matrix of distances to anti-ideal solution.

	SF1	SF2	SF3	SF4	SF5	SF6	SF7	SF8	F3
A1	0.00016	0.01703	0.00005	0.00003	0.00007	0.00004	0.00000	0.00000	0.00856
A2	0.00064	0.01346	0.00007	0.00018	0.00018	0.00004	0.00000	0.01040	0.03423
A3	0.00057	0.01346	0.00005	0.00000	0.00000	0.00010	0.00000	0.00000	0.00000
A4	0.00000	0.01703	0.00000	0.00003	0.00002	0.00000	0.00260	0.00260	0.00000
A5	0.00018	0.00757	0.00000	0.00000	0.00000	0.00000	0.00000	0.00000	0.00000
A6	0.00002	0.00000	0.00000	0.00000	0.00000	0.00000	0.00042	0.00042	0.00000
A7	0.00057	0.01346	0.00000	0.00001	0.00000	0.00001	0.01040	0.01040	0.03423
	SF9	SF10	SF11	SF12	SF13	SF14	SF15	SF16	Si-
A1	0.00109	0.00000	0.00109	0.00048	0.00014	0.00279	0.00051	0.00000	0.17899
A2	0.00436	0.00449	0.00436	0.00194	0.00102	0.00008	0.00203	0.00000	0.27829
A3	0.00000	0.00449	0.00436	0.00194	0.00006	0.00008	0.00203	0.00185	0.17025
A4	0.00109	0.00112	0.00109	0.00048	0.00006	0.00279	0.00051	0.00046	0.17289
A5	0.00109	0.00112	0.00109	0.00000	0.00006	0.00279	0.00051	0.00046	0.12195
A6	0.00017	0.00018	0.00017	0.00008	0.00006	0.00000	0.00008	0.00007	0.04103
A7	0.00436	0.00449	0.00436	0.00000	0.00102	0.00008	0.00203	0.00185	0.29538
